# A red-green photochromic bacterial protein as a new contrast agent for improved photoacoustic imaging^☆^

**DOI:** 10.1016/j.pacs.2022.100358

**Published:** 2022-04-16

**Authors:** Francesco Garzella, Paolo Bianchini, Alberto Diaspro, Aba Losi, Wolfgang Gärtner, Stefania Abbruzzetti, Cristiano Viappiani

**Affiliations:** a Dipartimento di Scienze Matematiche, Fisiche e Informatiche, Università di Parma, Italy; b Nanoscopy @ Istituto Italiano di Tecnologia, Genova, Italy; c DIFILAB, Dipartimento di Fisica, Università di Genova, Genova, Italy; d Institut für Analytische Chemie - Universität Leipzig, Leipzig, Germany

**Keywords:** Photochromic proteins, Bacterial phytochromes, Contrast agent, Time resolved photoacoustics

## Abstract

The GAF3 domain of the cyanobacteriochrome Slr1393 from *Synechocystis sp*. PCC6803, binding phycocyanobilin as a chromophore, shows photochromicity between two stable, green- and red-absorbing states, characterized by relatively high photoconversion yields. Using nanosecond-pulsed excitation by red or green light, respectively, and suitable cw photoconversion beams, we demonstrate that the light-modulatable photoacoustic waveforms arising from GAF3 can be easily distinguished from background signals originating from non-modulatable competitive absorbers and scattering media. It is demonstrated that this effect can be exploited to identify the position of the photochromic molecule by using as a phantom a cylindrical capillary tube filled with either a GAF3 solution or with an *E.coli* suspension overexpressing GAF3. These properties identify the high potential of GAF3 to be included in the palette of genetically encoded photochromic probes for photoacoustic imaging.

## 1 Introduction

Photoacoustic (PA) imaging of biological tissues relies on the acoustic detection of the pressure waves generated by non-radiative relaxation following light absorption by the sample. [1], [2], [3] The combination of optical excitation with ultrasonic detection offers advantages over optical detection because biological tissues are more transparent to sound than to light, thus allowing detection of signals originating deeper in the sample.

The contrast in photoacoustic imaging is based on the different absorption by tissue components such as hemoglobin in blood, other hemeproteins in tissues, melanin, endogenous metabolites, or specific dyes either added to the tissue or genetically encoded in the constituent cells. Discriminating between several different chromophoric compounds can be accomplished by multi-wavelength excitation and unmixing.

Contrast agents such as transgenic chromophores originally developed for fluorescence imaging provide less than optimal photoacoustic signal generation, given the low fraction of absorbed energy released as heat from these absorbers. [4], [5], [6] To overcome this issue, GFP-like non-fluorescent chromoproteins from Anthozoa species were proposed. [7], [8], [9].

More recently, bacterial phytochrome photoreceptors have been developed as optogenetic and imaging tools, characterized by absorption spectra covering the far red- near infrared portion of the electromagnetic spectrum, thanks to their ubiquitous biliverdin choromophore. [10], [11], [12] These receptors have several advantages for photoacoustic imaging since they have low fluorescence yields (typically 10^−2^-10^−3^) [13] and high molar absorption coefficients (85,000–110,000 M^−1^cm^−1^). [14], [15], [16] To further improve such applications such as contrast increase in photoacoustic imaging, reversibly switchable fluorescent proteins have been employed. [11], [17], [18].

Normally triggered by a cis-trans isomerization of the bound chromophore, photoswitchable proteins undergo a change in their absorption spectra under illumination with a specific wavelength. Lock-in detection of the light-driven modulation of the absorption properties was shown to strongly improve the contrast over the large background originating from non-modulated absorption of endogenous chromophores. [18], [19], [20] Application of this method to weakly or non-fluorescent photochromic probes leads to enhancement of the photoacoustic signal, as recently demonstrated for a bacterial phytochrome (bPhy), switching between a red- and a NIR-absorbing form. [21], [22], [23] Lifetime changes associated with light driven monomer-dimer equilibria of such proteins were also exploited to increase the contrast in photoacoustic imaging. [24].

Despite their demonstrated advantages, bacterial phytochromes suffer from their relatively large size that might interfere with several applications. bPhys request a combination of three sequentially arranged protein domains (a PAS, a GAF, and a PHY domain) as the chromophore binding domain (CBD) to guarantee / to maintain their spectral and functional features bringing the molecular mass of these proteins to > 60 kDa. To overcome this issue, smaller size (ca. 17 kDa molecular mass), red/near infrared photochromic proteins, termed sGPC2 and sGPC3, were engineered from the biliverdin-binding second GAF domain of the cyanobacteriochrome of *Acaryochloris marina* MBIC11017 cyanobacteria. [25] sGPC2 was recently exploited to simultaneously image *Escherichia coli* expressing sGPC2 and the larger bacterial phytochrome BphP1 from *Rhodopseudomonas palustris* [19] injected in mice in vivo. The photochromic properties of sGPC2 allowed a remarkable improvement in contrast-to-noise ratio in vivo over traditional PA imaging. [26].

We propose here a red-/green-switching cyanobacterial photochromic photoreceptor of ca. 18 kDa molecular mass [27] that expands the palette of small size, genetically encoded reporters for photothermal imaging.

**Fig. 1:**
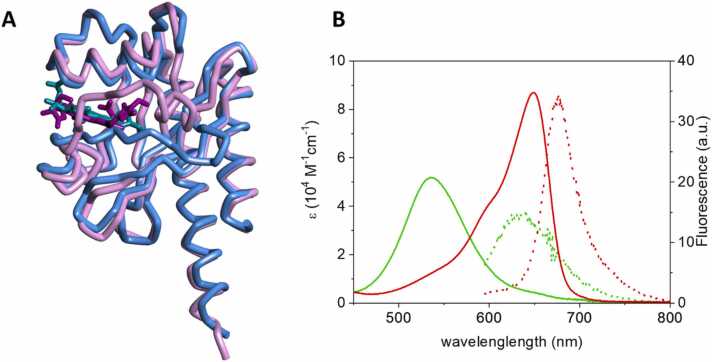
**A.** Superposition of the three-dimensional structures of the parental state (GAF3_R_, tube, light blue, PDB: 5dfx) [30] and of the photoproduct state (GAF3_G_, tube, magenta, PDB: 5m82) [30] of GAF3 from Slr1393 of *Synechocystis sp.* PCC6803 (in vivo assembled protein/chromophore). The phycocyanobilin chromophore is shown as sticks in cyan for GAF3_R_ and purple for GAF3_G_. **B.** Absorption coefficient (solid lines) and fluorescence emission (dotted lines) of the parental (GAF3_R_, red) and the photoproduct (GAF3_G_, green) states of GAF3 from Slr1393 of *Synechocystis sp.* PCC6803.

The third GAF domain (aa. 441–597, referred to as GAF3) of the protein encoded by the gene *slr1393* from the cyanobacterium *Synechocystis sp.* PCC6803 covalently binds phycocyanobilin (PCB) as the chromophore. GAF3 shows photochromicity and can be switched between a red-absorbing parental state (GAF3_R_, λ_max_ = 649 nm, Fig. 1A) and a green absorbing photoproduct state (GAF3_G_, λ_max_ = 536 nm, Fig. 1B) upon appropriate irradiation. The parental and the photoproduct species show absorption maxima separated by more than 100 nm and with reduced spectral overlap, especially in the red region of the spectrum (Fig. 1B). Using two independent methods, the quantum yield for the green-red conversion was estimated as ≈ 0.3, at least three times larger than that for the red-green conversion (≈0.08), [28] and significantly larger than that of canonical plant or bacterial phytochromes (typically ca. 0.15 or less). [13].

After photoexcitation a small portion of energy is emitted as fluorescence (Fig. 1B), with quantum yields Φ_F,R_ = 0.1 and Φ_F,G_ = 0.06 for GAF3_R_ and GAF3_G_, respectively. [28] The relatively weak fluorescence emission is an added value that has been exploited in fluorescence imaging. [28].

Thus, this probe combines interesting features such as high photochromic yield in both directions, low but appreciable fluorescence emission, and a very large spectral shift in the absorption of the two isomers.

Photoisomerization of the PCB chromophore is accompanied by an efficient heat release. Analysis of the photoacoustic signal revealed that after 650 nm excitation, about 76 ± 2% of the absorbed energy is released promptly (i.e. within ca. 50 ns from excitation) as heat from GAF3_R_ during the first steps of the R-G conversion. Similarly, about 71 ± 3% of prompt heat is released from GAF3_G_ upon 540 nm excitation ultimately leading to GAF3_R_. [27] It is worth mentioning that an additional transient, with lifetime at the limit of the time resolution, was detected in the photoacoustic experiment, in association with the GAF3_G_-to-GAF3_R_ photoconversion. [27] No similar transient was detected in association with the GAF3_R_-to-GAF3_G_ photoconversion. In this work we further investigate the GAF3_R_-to-GAF3_G_ transition using a setup with improved time resolution (about 20 ns).

The high molar absorption coefficient of the compound (ε(649 nm)= 87000 M^−1^cm^−1^ for GAF3_R_; ε(540 nm)= 51500 M^−1^cm^−1^ for GAF3_G_) [29] and the efficient heat release make GAF3 a promising probe for perspective applications in photothermal imaging.

Although the spectral range in which GAF3 absorbs overlaps with a region of absorption of endogenous compounds, the photochromic behavior is expected to allow contrast improvement in photoacoustic imaging due to its photo-converting properties, as demonstrated for other bacterial phytochromes. [21], [22], [23], [26] Taking advantage of the large spectral shift between GAF3_R_ and GAF3_G_, in this work we explore the possibility of distinguishing the photoacoustic signals for this photochromic protein from background signals arising from absorbing media, and in the presence of intense light scattering.

## 2 Materials and methods

Brilliant Black BN (BBBN) was from Sigma-Aldrich.

### 2.1 Protein expression and purification

The expression and purification, as well as the two-plasmid approach are described in detail in [31], [32]. In brief, Slr1393g3 was produced as an N-terminally His-tagged protein in *E. coli* BL21(DE3) using a pET30 expression vector. The recombinant protein was expressed by a two-plasmid transformation/ expression protocol allowing in vivo assembly and was purified as previously described. [28], [31] The buffer used in the present studies was a 10 mM phosphate buffer, pH = 7.5.

### 2.2 Time resolved photoacoustics

The principles of PA signal generation have been described. [33], [34] The PA signals were collected with a dedicated experimental layout, obtained by modifying a previously reported setup. [27] Nanosecond excitation of the photoacoustic signal was achieved with the tunable output of the optical parametric oscillator of an Integra system (GWU-Lasertechnik Vertriebsges. mbH, Germany). Depending on the probed species, the output at either 540 nm (for probing GAF3_G_) or 650 nm (for probing GAF3_R_) was selected. The repetition rate was kept at 5 Hz. The laser pulse energy was kept in the linear signal range and was monitored by a laser energy meter (Laser Probe) during the data acquisition.

PA signals were detected using a Panametrics, V-103 piezoelectric transducer (1 MHz). After amplification (Panametrics model 5662 ultrasonic preamplifier,0.5–5 MHz, 54-dB gain) the signal was fed into a digital oscilloscope (LeCroy 9370) operated at 500 MS/s in a 10 μs time window, and finally transferred to a personal computer for further elaboration. Each PA signal was the average of 100 shots. For deconvolution analysis, BBBN was used as a photocalorimetric reference compound. [35].

The quartz sample cuvette was mounted into a temperature-controlled sample holder (TASC 300, Quantum Northwest, Spokane, WA, USA), which ensured a temperature stability of better than 0.02 °C inside the solution. A thin layer of vacuum grease was used to optimize mechanical and acoustic coupling between transducer and cuvette. The cuvette is never removed from the holder during the experiments. Care was taken not to perturb the transducer-cuvette arrangement when changing samples. [36].

The laser beam was shaped with a 270 µm vertical slit (corresponding to an acoustic transit time of roughly 200 ns), [33] providing a time resolution of about 20 ns after deconvolution of the photoacoustic signal against a photocalorimetric reference compound. [37], [38] In our previous work on GAF3 we employed a 1 mm wide slit, resulting in a 3-fold higher acoustic transit time, which limited time resolution to ca. 50 ns. [27] Deconvolution analysis of PA signals was performed using the software Sound Analysis (Quantum Northwest, Spokane, WA, USA).

### 2.3 Generation of GAF3_G_ and GAF3_R_ states

For experiments in homogeneous solutions, photoconversion from GAF3_G_ to GAF3_R_ state was achieved by the cw emission at 514 nm of a multiline argon ion laser (JDS Uniphase 150 mW all lines). The output was set to obtain 15 mW at 514 nm. The reverse photoconversion was induced with the combined use of the 633 nm output of a HeNe laser (30 mW) and the 670 nm output of a diode laser (3 mW). Full photoconversion of the sample was achieved in both cases within 1 min. The selection of the laser line was obtained using two shutters with the shutter drivers operating in opposite mode (Uniblitz, Vincent Associates Inc., Rochester, NY, USA).

During collection of the photoacoustic signals from GAF3, in order to prevent accumulation of the photoproducts generated by the pulsed excitation, the sample was kept under cw illumination with either the green 514 nm laser (to preserve the GAF3_R_ state), or with the 633/670 nm laser (to keep the system in the GAF3_G_ state). The sample was stirred with a spin bar to ensure homogeneity of the molecular species in the entire volume. The photoconversion beams entered the cuvette at right angle to the pulsed laser beam, from the side opposite the piezoelectric transducer. Absorption of the light of the photoconversion beams from the transducers’ surface results in no detectable signal, thus this introduced no systematic errors.

### 2.4 Deconvolution and separation of enthalpic and volumetric contributions

Preprocessing of the PA signals requires subtraction of the baseline (i.e. a signal collected with the laser beam blocked), from the signal obtained when the laser beam hits the sample. Signals were then normalized for sample absorbance at the excitation wavelength and for laser pulse energy. Signals for samples (GAF3_R_ or GAF3_G_) were compared to an absorbance- and laser energy-normalized Brilliant Black BN (BBBN) reference signal measured under identical temperature conditions. Reference and sample signals were normalized to the maximum of the normalized reference signal before performing deconvolution analysis. [37], [38] A detailed description of the signal processing and analysis can be found in the literature. [34].

Deconvolution was performed using the program Sound Analysis (Quantum Northwest, Spokane, WA, USA). Signals were fitted by reconvolution of the waveform for the photocalorimetric reference compound (BBBN) with an exponential decay or a sum of two exponential decays, depending on temperatures. [37], [38] From deconvolution we retrieved the pre-exponential factors (φ_i_) and lifetimes (τ_i_).

$$S\left(t\right)=\overset{2}{\underset{i=1}{\sum }}\frac{\varphi_{i}}{\tau_{i}}\exp\left(-\frac{t}{\tau_{i}}\right)$$

Pre-exponential factors φ_i_ contain enthalpic and volumetric contributions. Separation of these terms was achieved by collecting the PA signals of GAF3_R_, GAF3_G_, and BBBN as a function of the thermoelastic parameter C_p_ρ/β, exploiting its strong temperature dependence. [33], [34] In the above expression, C_p_ is the specific heat at constant pressure, ρ is the density, and β is the cubic thermal expansion coefficient of the solution. The values of C_p_ρ/β as a function of temperature for the buffer used in these studies has been determined by a comparative method, using the known temperature dependence of C_p_ρ/β for water. [33] The investigated temperature range was between 5 and 25 °C. The pre-exponential factors φ_i_ were plotted as a function of C_p_ρ/β using the linear relation, derived from an euristic approach: [33], [34].

$$\varphi_{i}=\alpha_{i}+\frac{\Delta V_{i}}{E_{\lambda }}\frac{C_{p}\rho }{\beta }$$

In this equation, E_λ_ is the molar energy of incident photons and β is the isobaric volume expansion coefficient. From the linear plot of φ_i_E_λ_ vs C_p_ρ/β we estimated for each transient the fraction of absorbed energy released as heat (from the intercept, α_i_) and the structural volume change (from the slope, ΔV_i_). Molar structural volume changes, ΔV_R,i_, were then calculated from observed volume changes according to ΔV_R,i_ = ΔV_i_/Φ_i_. where Φ_i_ is the photoisomerization quantum yield.

Further data analysis was performed using OriginLab Pro (OriginLab Corporation, Northampton, MA 01060, USA) and Matlab (The MathWorks, Inc., USA).

### 2.5 Photoacoustic spectra

Photoacoustic spectra were reconstructed by collecting PA waveforms for GAF3_R_ and GAF3_G_ solutions at room temperature as a function of the pulsed laser excitation wavelength in the range 450–670 nm. The signals were corrected for the baseline and normalized for the laser pulse energy. The amplitude of the first positive oscillation of the waveform was taken as a measure of the signal intensity.

### 2.6 Capillary tube experiments

**Fig. 2:**
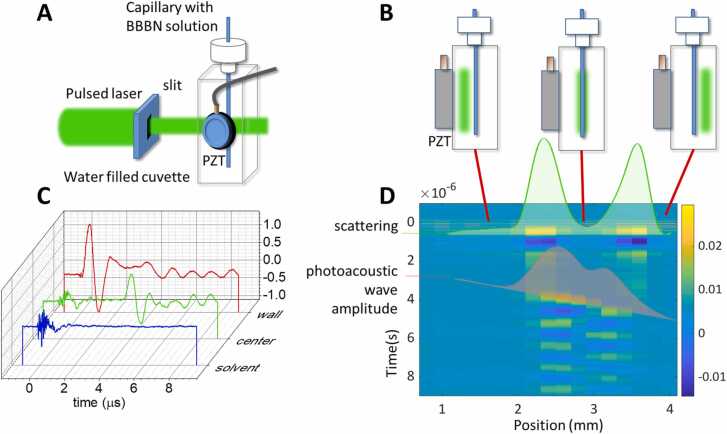
A. Schematic of the cuvette with the capillary tube, the laser beam, and the piezoelectric transducer (PZT). B. Front view of the cuvette with the capillary and the laser beam at three different positions (passing to the left, through the center, or to the right of the capillary). C. Representative waveforms collected with the laser beam passing through the solvent (blue), the center of the capillary (green), or the capillary wall (red). The capillary contained a BBBN solution with absorbance 0.5 (1 cm pathlength) at the excitation wavelength. The oscillation starting at time ∼0 s at all positions is due to the scattered light absorbed by the transducer’s surface. The oscillation starting at about 3.5 μs in the green waveform is due to the signal generated upon absorption by the solution inside the capillary. The scattering signal becomes very large when the laser beam hits the capillary wall (red) and a smaller amplitude oscillation is appreciable at about 4 μs, due to absorption of the laser beam by the solution inside the capillary. To emphasize the scattering signal, these data were collected without the aluminum foil between cuvette and transducer, which was present in all other experiments with the capillary tube. D**.** Contour plot of the PA signals collected at different beam positions inside the cuvette. The signal due to laser scattering is observed at t ∼ 0 s at all beam positions. The width of this signal (green solid line) provides an estimate of the external diameter of the capillary (1.6 ± 0.2 mm FWHM), consistent with the direct measurement using a caliper. Due to the finite speed of sound (∼1500 m/s in water), the signal coming from absorption by the solutions inside the capillary is observed at increasing delay when the cuvette is scanned, and the distance between the transducer and the beam increases. The red shaded area reports the amplitude of the acoustic wave generated inside the capillary, whose time-position dependence nicely reproduces the speed of sound in the solvent (∼1500 m/s). The width of the red curve (FWHM) is 1.0 ± 0.2 mm, identical to the one measured through an optical microscope with a calibrated ruler.

A 1 cm × 1 cm plastic cuvette (Sigma-Aldrich) with four optical windows was inserted into the PA sample holder. A piece of aluminum foil was placed between the cuvette and the piezoelectric transducer to reflect stray light from the pulsed laser beam and to prevent generation of a large amplitude acoustic wave by direct absorption of photons by the transducer’s surface. Thin layers of vacuum grease were put between the cuvette, the aluminum foil, and the transducer. A small cylindrical glass capillary tube (outer diameter 1.6 mm, inner diameter 1.0 mm) sealed at the bottom was mounted vertically in the cuvette (Fig. 2A). The capillary contained the solution of interest (a BBBN solution, a GAF3 solution, or a suspension of *E. coli* expressing GAF3). Depending on the experiment, the cuvette where the capillary was immersed was filled; i) with water, as an ideal case with no competitive absorption or scattering; ii) with a BBBN solution, to simulate a case where competitive absorption occurs; iii) with an *E. coli* bacterial suspension to simulate a case where a scattering medium is present.

The position of the beam (shaped by the 270 µm vertical slit) was scanned horizontally through the cuvette (Fig. 2B) using a micro-positioning translation stage. PA signals were collected at positions separated by 200 µm steps. Fig. 2C shows selected PA signals for a capillary containing BBBN and immersed in water. When the beam is passed through the solvent without hitting the capillary (blue signal), essentially no photoacoustic signal is observed. When the beam passes through the center of the capillary (green curve), a clearly visible PA signal is detected at ca. 3.5 μs. When the beam hits the capillary wall (red waveform), a large signal is observed at time 0, due to intense scattered light hitting the transducer’s surface. A smaller size PA signal is also evident at ca. 4 μs, due to partial overlap of the laser beam with the solution inside the capillary. The position dependent PA signals are shown in Fig. 2D using a contour plot.

Photoconversion beams for these experiments were obtained by coupling a frequency-doubled cw Nd:YAG laser (100 mW) or a high power LED (central wavelength 670 nm, 50 mW) to a fiber bundle. The beam output entered the cuvette at right angle to the pulsed laser beam. The beam completely filled the volume of the capillary.

## 3 Results and discussion

### 3.1 Time resolved heat release and volume changes

In the current work we have taken advantage of a slightly higher time resolution of the time resolved photoacoustic setup (ca. 20 ns for the laser beam width of 270 µm and the 1 MHz transducer employed in the present experiments), to re-evaluate the heat release and volume change associated with the photoconversion processes we have previously determined with a lower time resolution setup (ca. 50 ns). [27] This section provides fundamental properties of the photochromic photoacoustic signals that will be exploited in the following sections to enhance contrast.

**Fig. 3:**
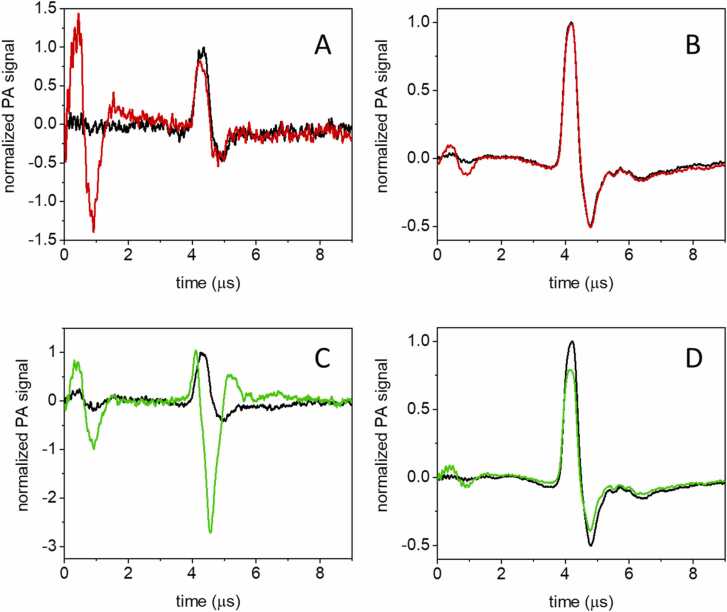
PA signals for GAF3_R_ (A) and GAF3_G_ at 5 °C (C) and 20 °C (GAF3_R_: B, GAF3_G_: D). The top panels report a comparison between the laser pulse energy- and absorbance-normalized signals from BBBN (black) and GAF3_R_ (red) at 5 °C (A) and at 20 °C (B). Excitation wavelength was 650 nm. Bottom panels report a comparison between the laser pulse energy- and absorbance-normalized signal from BBBN (black) and GAF3_G_ (green) at 5 °C (C) and at 20 °C (D). Excitation wavelength was 540 nm. Deconvolution analysis of the signals is reported as Supporting Information Fig. S1.

Fig. 3 shows the laser pulse energy and absorbance normalized PA signals for GAF3_R_ (A, B, red curves) and GAF3_G_ (C, D, green curves) at 5 °C (A, C) and 20 °C (B, D). The reference signals from BBBN (black curves) are shown for comparison.

The signals for GAF3_R_ and BBBN at 20 °C (Fig. 3B) present a comparable amplitude, an indication that the heat release upon photoexcitation is similar. Their time profile is also similar, but the slight shift of the GAF3_R_ signal hints to a transient occurring at the limit of the time resolution of the setup. The presence of a second transient becomes visible at 5 °C (Fig. 3A), where the contribution from structural volume changes to the PA signal is comparatively more relevant. The shape of the waveform suggests the presence of a contraction in the tens of nanoseconds range, which is responsible for the faster decay of the first positive oscillation, crossing the horizontal axis to the left of the reference waveform, at ca. 4.5 μs. This second transient was not detected in our previous investigation, due to the lower resolution (∼50 ns) of the setup used in that case, where a 1 mm wide slit was used. [27].

The signal from GAF3_G_ at 20 °C (Fig. 3D) is lower in amplitude than the signal of the reference compound. The shape indicates that a transient of negative amplitude with lifetime in the tens of nanoseconds follows a fast, sub-resolution positive signal. The negative signal becomes very evident at 5 °C (Fig. 3C).

We note that at t = 0 s, an intense oscillation is observed, due to absorption of light by the surface of the transducer, coming from fluorescence emission by GAF3_G_ or GAF3_R_, excited by the laser pulse, and, to a smaller extent, by scattered light. The latter contribution is evident also in the signals collected for the reference compound.

Deconvolution analysis of signals for GAF3_G_ and GAF3_R_ allows to retrieve the fractional amplitudes of the transients at each temperature. The best fit of the transients was obtained with a single exponential decay, with lifetime below the time resolution (∼20 ns) at the higher temperatures, or with the sum of two exponential decays for temperatures below 15 °C. Representative fits are reported in Supporting Information (Fig. S1). In the lower temperature range, it was possible to determine the lifetime of the second, slower transient occurring in the 20–100 ns time range. The estimate of the fractional amplitudes is affected by the short lifetime of the resolvable phase, occurring at the limit of the time resolution of the setup. Under these conditions, amplitudes and lifetimes show correlation and cannot always be retrieved reliably. Thus, low temperature data, where the lifetime of the second transient become longer, usually afforded more reliable parameters.

**Fig. 4:**
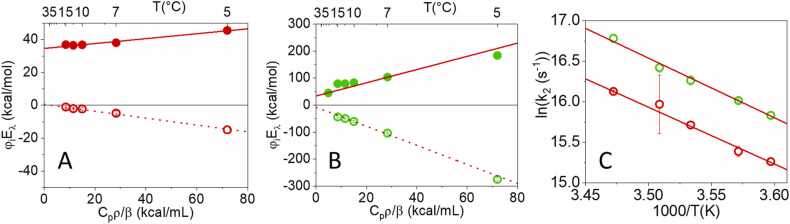
Plot of the energy content of each transient (φ_i_E_λ_) as a function of C_p_ρ/β for GAF3_R_ (A) and GAF3_G_ (B). Filled circles, φ_1_E_λ_, open circles, φ_2_E_λ_. Red lines are the linear fits to the data. C**.** Arrhenius plot for k_2_ = 1/τ_2_ for GAF3_R_ (red open circles) and GAF3_G_ (green open circles). Lifetimes τ_2_ were retrieved only at temperatures below 15 °C.

The thermodynamic information contained in the fractional amplitudes of the exponential decays is extracted from Fig. 4 (panels A and B), where the energy content of each transient (φ_i_E_λ_) is plotted as a function of C_p_ρ/β. From the intercepts and slopes retrieved by the linear fits the fraction of absorbed energy released as heat (α_i_) and the molar structural volume change (ΔV_R,i_) were calculated (see Table 1).

**Table 1:** Fractions of absorbed energy released as heat and molar structural volume changes. Activation energies for the slow process 2 were determined from the linear Arrhenius plot.

	α_1_	ΔV_R,1_ (mL/mol)	α_2_	ΔV_R,2_ (mL/mol)	ln (A)	E_a_ (kcal/mol)
GAF3_R_	0.79 ± 0.01	1.9 ± 0.1	0.01 ± 0.01	-2.6 ± 0.3	40.5 ± 0.5	13.9 ± 0.3
GAF3_G_	0.64 ± 0.05	8 ± 1	-0.15 ± 0.08	-11.7 ± 0.8	42 ± 1	14.6 ± 0.6

In keeping with our previous determination, for both species, an expansion accompanies the fast E-Z photoisomerization (labelled as process 1 in the kinetic analysis, with lifetime below ∼10 ns). The reaction volume is larger for the GAF3_G_ to GAF3_R_ conversion.

The α_1_ values are in keeping with our previous determinations (0.76 ± 0.02 for GAF3_R_ to GAF3_G_ 0.71 ± 0.03 for GAF3_G_ to GAF3_R_), [27] the small differences being possibly due to the better resolution of the present work. Thus, the energy content of the first reaction intermediate is comparable to our previous estimate.

The relatively large contraction associated with the second transient (for the GAF3_G_ to GAF3_R_ conversion), labelled as 2 in the kinetic analysis, is consistent with our previous findings. However, thanks to the improved time resolution, a smaller amplitude volume change, with lifetime in the 10–100 ns range, was detected also for the GAF3_R_ to GAF3_G_ photoconversion. Interestingly, the heat release (enthalpic change) for the slower processes is zero within the experimental error. The activation energies for the contractions are estimated from the linear Arrhenius plots in Fig. 4C. The values reported in Table 1 are similar and indicate that a substantial barrier must be overcome in this kinetic step.

### 3.2 Photochromic properties and photoacoustic spectra

**Fig. 5:**
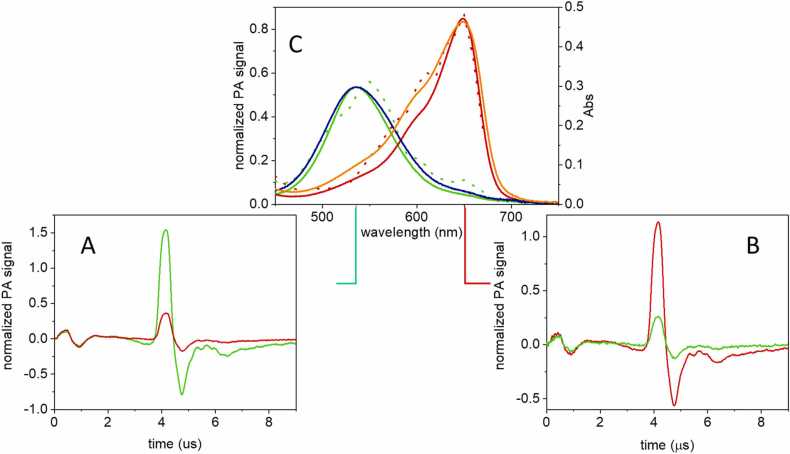
A. Energy-normalized PA signals for a GAF3 solution excited at 540 nm (green, GAF3_G_; red, GAF3_R_). B. Energy normalized PA signals for a GAF3 solution excited at 650 nm (green, GAF3_G_; red, GAF3_R_). C. PA signal amplitude for GAF3_G_ (green dotted line) and GAF3_R_ (red dotted line) as a function of excitation wavelength. Signal amplitudes were estimated from the first positive oscillation (at ∼ 4 μs) and were normalized for laser pulse energy. For comparison, the corresponding absorption spectra are reported as solid green and solid red lines. The solid dark blue and solid orange lines show the plots for 1–10^-A^, calculated for the absorption spectra of GAF3_G_ and GAF3_R_, respectively. T = 20 °C. [GAF3]= 5 μM.

The photochromic properties of GAF3 reported in the previous section can be exploited in PA detection to increase the contrast in sensing the compound in the presence of background-absorbing media. Fig. 5 compares the PA signals observed for GAF3_G_ and GAF3_R_ after excitation in the main absorption bands of the two species. Fig. 5A demonstrates that upon pulsed excitation at 540 nm and concomitant 633/670 nm cw excitation, a large and stable PA signal is observed for GAF3_G_ (green curve). The signal is strongly reduced (∼4-fold) when GAF3_R_ is generated upon cw photoconversion with 514 nm light, with a ratio in keeping with the change in molar absorption coefficient at this wavelength. [28] The change in amplitude is similar (∼4.5-fold, Fig. 5B) when pulsed excitation at 650 nm is used, and GAF3_R_ (originally obtained with 514 nm excitation) is converted to GAF3_G_ with 633/670 nm cw illumination. The smaller than expected change in amplitude under 650 nm excitation, on the basis of the absorption coefficients of GAF3_R_ and GAF3_R_ (∼15-fold), is attributed to a difficulty in obtaining full conversion under the employed experimental conditions.

In order to correlate the PA signals reported in Fig. 5A and B with the photochromic properties of GAF3, we have measured PA action spectra for GAF3_R_ and GAF3_G_. As in the previous experiments, PA signals for GAF3_G_ (GAF3_R_) were collected under 633/670 nm (514 nm) cw illumination. Fig. 5C reports the PA signal intensity measured as a function of the pulsed excitation wavelength for GAF3_G_ (green solid curve) and for GAF3_R_ (red solid curve). The shape of the PA spectra roughly follows that of the absorption spectra, reported as the dotted lines, confirming that the observed signals arise from the two molecular species. However, PA signal amplitude is proportional to 1–10^-A^, thus a better agreement with the PA spectra is observed if 1–10^-A^ for GAF3_G_ (dark blue curve) or GAF3_R_ (orange curve) are plotted. In particular, the orange curve in Fig. 5C perfectly overlaps the PA spectrum for GAF3_R_. The agreement is not perfect for GAF3_G_. This effect has been attributed to wavelength-dependent incomplete conversion of GAF3 in the range where absorption spectra strongly overlap, which may reflect also the larger green to red photoconversion yield.

### 3.3 Photochromic signals of GAF3 in presence of competitive absorbers

**Fig. 6:**
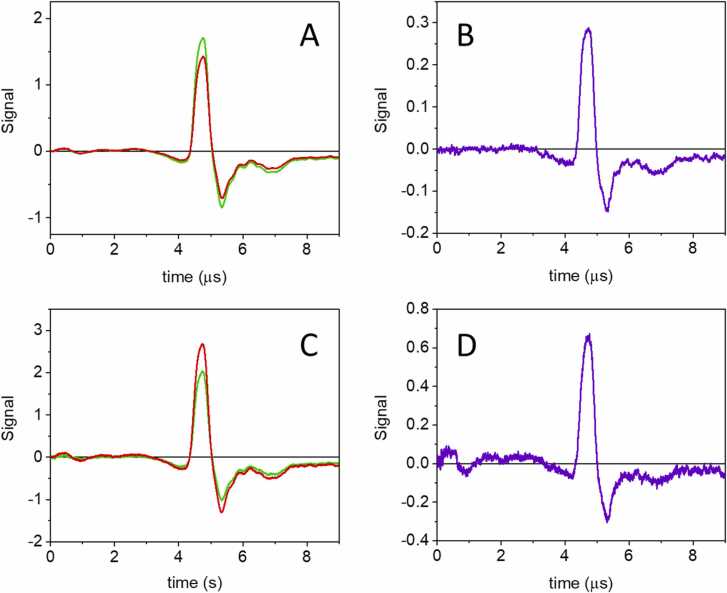
PA signals for GAF3_R_ (red curves) and GAF3_G_ (green curves) solutions, after excitation at 540 nm (A) or 650 nm (C). The solutions contained also BBBN at a concentration corresponding to an absorbance of 0.5 at 540 nm and 0.75 at 650 nm. Difference of PA signals at 540 nm (GAF3_G_ minus GAF3_R_, B) and at 650 nm (GAF3_R_ minus GAF3_G_, D), showing a strong contrast even in the presence of a competitive absorber.

The photochromic modulation of the PA signal amplitude generated by GAF3 allows to distinguish its contribution in the presence of absorbing, non-photochromic compounds. This mimics the situation encountered in tissues, where other absorbers are present, most likely not showing photochromicity. As the simplest case, we have acquired the PA signal of a GAF3 solution exciting at 650 nm and 540 nm for GAF3_R_ and GAF3_G_, in the presence of competitive absorbance by a co-solute, BBBN, at increasing concentrations. Absorbance of GAF3_R_ at 650 nm was 0.22, and that of GAF3_G_ was 0.14 at 540 nm. The range of competitive absorbance by BBBN was 0–0.5 at 540 nm, and 0–0.75 at 650 nm. Fig. 6 compares selected PA signals for GAF3_R_ and GAF3_G_ after excitation at 540 nm (A) or 650 nm (C) for the highest BBBN concentration investigated, corresponding to an absorbance of 0.5 at 540 nm and 0.75 at 650 nm. The extent of the change in PA signal intensity (contrast) can be evaluated by subtracting the two waveforms (the difference signals are shown in panels B and D). Although the non-modulatable PA signal is quite intense, the modulatable fraction is clearly evident in the plots, and gives rise to a reasonably high contrast. As observed previously, due to incomplete conversion, the ratio between signals in D and B is smaller than the ratio between absorption coefficients of GAF3_R_ and GAF3_G_ at the excitation wavelengths.

### 3.4 Photochromic signals of GAF3 in presence of scattering media

**Fig. 7:**
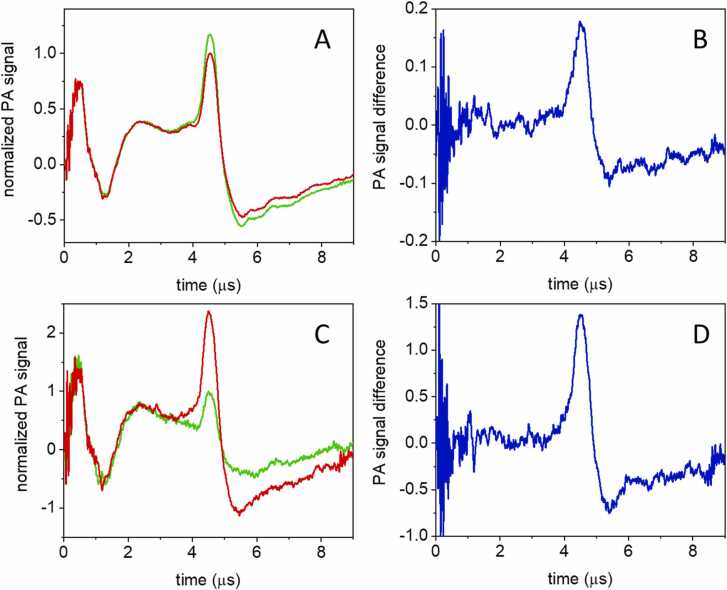
PA signals for a suspension of *E. coli* overexpressing GAF3 after excitation at 540 nm (A) or 650 nm (C) for GAF3_R_ (red signals) and GAF3_G_ (green signals). After baseline subtraction and laser pulse energy normalization, signals were normalized to the lower amplitude waveform (GAF3_R_ in A and GAF3_G_ in C). In panels B and D we have evaluated the extent of change in PA signal intensity by subtracting the two waveforms.

As a next step, we investigated the photochromic signals for GAF3-overexpressing *E. coli* suspensions, where intense light scattering occurs. The photochromic protein is fully functional in the bacteria thanks to the two-plasmid transformation/expression protocol we have used (See Materials and Methods). For this suspension the optical density at 650 nm is around 0.6 and is mostly due to light scattering. Fig. 7 shows the PA signals for a suspension of *E. coli* overexpressing GAF3 after excitation at 540 nm (A) or 650 nm (C) for GAF3_R_ (red signals) and GAF3_G_ (green signals). The intensity of the signal of GAF3_R_ in A is much larger than expected. We attribute this to the presence of a competitive, non modulatable species present in the bacterial suspension, absorbing at 540 nm. On the contrary, the GAF3_G_ signal in panel C is consistent with expectations, and does not suggest the presence of additional competitive absorbers. As previously observed for the data in Fig. 6, the contrast can be evaluated by subtracting the two waveforms (panels B and D). The contrast is much better for the red absorbing species exploiting the 650 nm absorption band. Again, due to incomplete conversion, the ratio between signals in D and B (∼7) is not exactly matching the ratio between absorption coefficients of GAF3_R_ and GAF3_G_. at the excitation wavelengths.

### 3.5 Photochromic photoacoustic signals from a capillary tube

**Fig. 8:**
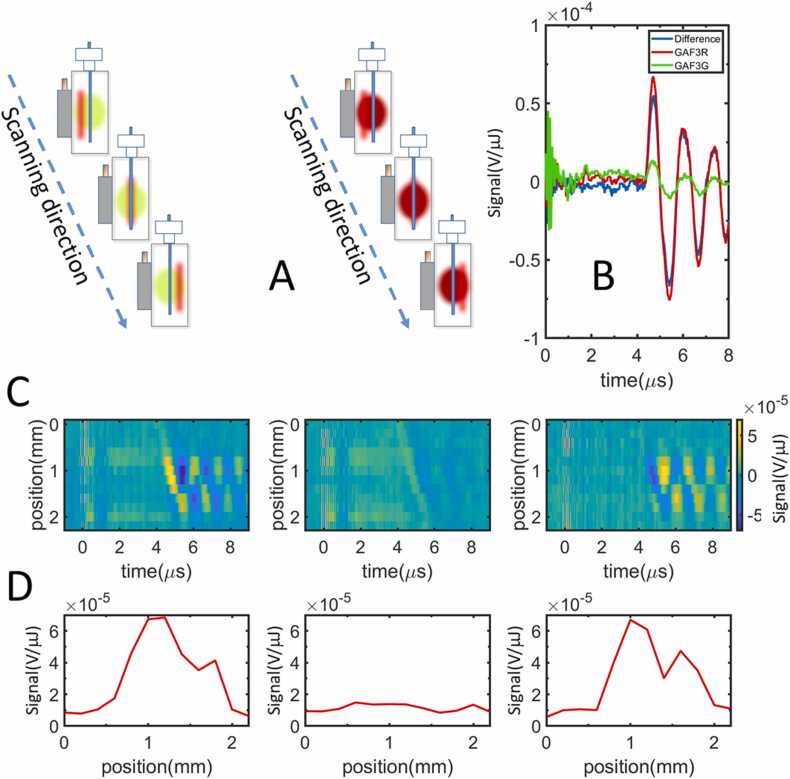
Capillary tube experiment for a 5 μM GAF3 solution. The cuvette is filled with deionized water (B, C, and D). The competitive absorbance of the BBBN solution was 0.75 (1 cm pathlength) at 650 nm, where excitation was performed. A. Schematic of the geometrical arrangement of the pump beam (vertical slit), the photoconversion beam (red or green circles) and the capillary tube at different pump beam scanning positions for GAF3_R_ (left) and GAF3_G_ (right). B. PA signals collected at the center of the capillary for GAF3_R_ (red), GAF3_G_ (green) and the difference between the signals (blue). C. Contour plot of the PA signals as a function of time and position of the beam in the cuvette for GAF3_G_ (left), GAF3_R_ (center) and difference between the two signals (right). D**.** Amplitude of the first positive PA oscillation for GAF3_R_ (left), GAF3_G_ (center) and difference between the two signals (right) as a function of the excitation beam position inside the cuvette.

For future imaging applications, it is important to establish the capability of the photochromic photoacoustic signal to provide the spatial distribution of GAF3. As a straight forward, simple system with a spatial photochromic protein distribution, we have filled a capillary tube (inner diameter ca. 1 mm) with a GAF3 solution and inserted the loaded capillary vertically into a water filled cuvette (see Fig. 2). The equally vertically positioned, pulsed 650 nm beam was scanned horizontally across the cuvette (in the presence of the proper cw beam to obtain either GAF3_R_ or GAF3_G_, Fig. 8A), and the resulting PA signals were collected. Fig. 8B reports selected signals collected for GAF3_R_ and GAF3_G_ under 650 nm excitation, at the center of the capillary tube, along with their difference. From simple visual inspection it is evident that the photoacoustic wave for GAF3_R_ is larger than the one for GAF3_G_. Similar results were obtained under 540 nm excitation, the only difference being an overall smaller amplitude of the PA signals due to lower absorbance at this wavelength. Fig. 8C reports the contour plot representation of the signals for GAF3_G_ and GAF3_R_ collected for the beam at different positions across the cuvette. As already observed for Fig. 2D, the arrival time of the acoustic wave generated by protein absorption inside the capillary becomes longer as the beam is scanned through the absorbing region. Plotting the amplitude of the first maximum of the acoustic wave for GAF3_R_ as a function of the beam position (Fig. 8D left) clearly shows an increase when the beam hits the solutions inside the capillary tube, which allows to easily identify the position of the capillary. For GAF3_G_ (Fig. 8D center) the signal is much smaller. The difference between the two signals reported in the right panel of Fig. 8D provides an estimate of the contrast in the identification of the spatial distribution of GAF3. It is worth noting that the signal becomes smaller near the center of the capillary, possibly due to optical interference caused by the curved capillary surface.

**Fig. 9:**
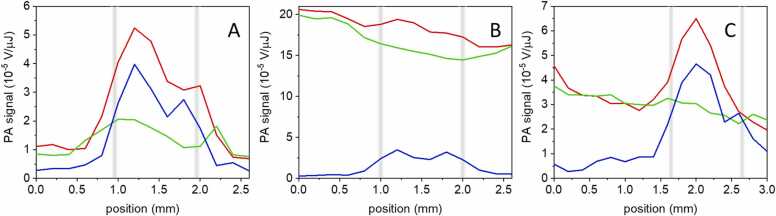
*E. coli* suspension overexpressing GAF3 in a capillary tube. Amplitudes of the first positive PA oscillation for GAF3_R_ (red), GAF3_G_ (green) and the difference between the two signals (blue) as a function of the excitation beam position inside the cuvette. Excitation at 650 nm. A. capillary tube immersed in water. B. capillary tube immersed in a BBBN solution with absorbance 0.75 at 650 nm. C. capillary tube immersed in an *E. coli* suspension. The position of the capillary was slightly different in each experiment. The vertical bars are visual aids to identify the position of the capillary walls.

We have next placed in the capillary an *E. coli* suspension overexpressing GAF3 and tested the capability to retrieve the spatial information in the photochromic PA signal when the capillary is immersed in water, in a BBBN solution (to model competitive absorption), or in an *E. coli* suspension with OD (650 nm) = 0.6 (to model the presence of a non-absorbing scattering medium). While pulsed excitation at 540 nm does not lead to satisfactory results (Supporting Information, Figs. S2, S4, and S6), excitation at 650 nm can retrieve the position of the capillary under all tested conditions, as shown in Fig. 9. (see Supporting Information Figs. S3, S5, and S7 for detailed plots).

Fig. 9 shows that the photochromic PA signal enables identifications of the spatial distribution of the protein (within the cylindrical capillary tube phantom) in the presence of competitive absorbance and scattering media. When the capillary is immersed in water, the position of the *E. coli* suspension overexpressing GAF3 is easily detected regardless of the photochromic behavior (Fig. 9A). However, in the presence of competitive absorption (Fig. 9B) or a non-absorbing scattering medium (Fig. 9C), the position of the capillary becomes evident only when the non-modulated background is suppressed thanks to the change in PA signal coming from GAF3_R_ and GAF3_G_. The double peak feature at the center of the capillary is due to optical artifacts due to the curvature of the capillary walls.

A contrast on the order of 5 is observed in all cases, with an efficient background signal suppression in panels B and C. In particular, when competitive background absorption is present (panel B) we were able to reject a non modulatable PA signal that was ∼4x larger than the signal from GAF3. In all cases a contrast-to-noise ratio of about 15 was obtained in the center of the capillary. It is worth noting that the capillary phantom was placed at ∼5 mm distance from the cuvette surface hit by the incoming laser. Although qualitative in nature, this information suggests that the contrast can be exploited to retrieve the spatial information at mm-deep positions inside the object under investigation.

## 4 Conclusions

The photoacoustic signal generated by photoexcitation of the photochromic protein GAF3 can be tuned by selecting the red or the green absorbing state, through irradiation with green or red light, respectively. The photochromic PA signal from GAF3 is clearly distinguished from competing absorbing species by enhancing the contrast and thus rejecting the background signals.

Using a capillary tube filled with a GAF3-containing solution as a phantom, we demonstrate that it is possible to identify the position of the tube through 1-D scanning of the laser beam across the medium into which the capillary is placed. Thanks to the photochromic behavior, the contrast can be enhanced, and background signal suppression is effectively achieved in the case of absorbing media and for non-absorbing, scattering media, without loss of spatial resolution. Interestingly, the photochromic PA signals recorded after red light excitation were found to be stronger than those originating from the green absorbing form.

GAF3, even as an isolated protein, binds PCB as a chromophore and provides all phytochrome-typical features of absorption and kinetics. Although this chromophore is not ubiquitous, in vivo binding of PCB to GAF3 can be obtained using a two-plasmid approach in *E. coli*, [28], [31] thus bypassing the need for external chromophore supplementation. While not in the ideal spectral transmission region, the extent of the red-green photochromism of GAF3 (with >100 nm spectral shift) provides an additional signal that may be useful in multispectral photochromic PA imaging.

As further advantage, the combination of large absorption coefficients and high photoconversion yields warrants very fast photoswitching of the protein between the two states. This feature is expected to reduce acquisition times in perspective multi-wavelength imaging. Moreover, the photoproduct state is remarkably stable in the dark.

Finally, the modest, but appreciable, fluorescence yield serves well as a local marker to be exploited in correlative microscopy. Further developments to test these photochromic proteins in PA imaging setups and extension of the two plasmid approach to eukaryotic cells will assess the whole potential of this photochromic probe for biological applications.

## Declaration of Competing Interest

The authors declare no competing interests.
