# Supplementary material for: A red-green photochromic bacterial protein as a new contrast agent for improved photoacoustic imaging
Source: Photoacoustics. 2022 Apr 16;26:100358. doi: 10.1016/j.pacs.2022.100358 (PMC9152790; doi:10.1016/j.pacs.2022.100358)
Supplement: Supplementary file 1 — Supplementary material [file mmc1.docx]

**Supporting Information**

**A red-green photochromic bacterial protein as a new contrast agent for improved photoacoustic imaging**

Francesco Garzella^1,2^, Paolo Bianchini^2^, Alberto Diaspro^2,3^, Aba Losi^1^, Wolfgang Gärtner^4^, Stefania Abbruzzetti^1^, Cristiano Viappiani^1^

^1^Dipartimento di Scienze Matematiche, Fisiche e Informatiche, Università di Parma

^2^Nanoscopy @ Istituto Italiano di Tecnologia, Genova, Italy

^3^DIFILAB, Dipartimento di Fisica, Università di Genova, Genova, Italy

^4^Institut für Analytische Chemie - Universität Leipzig, Leipzig, Germany

**
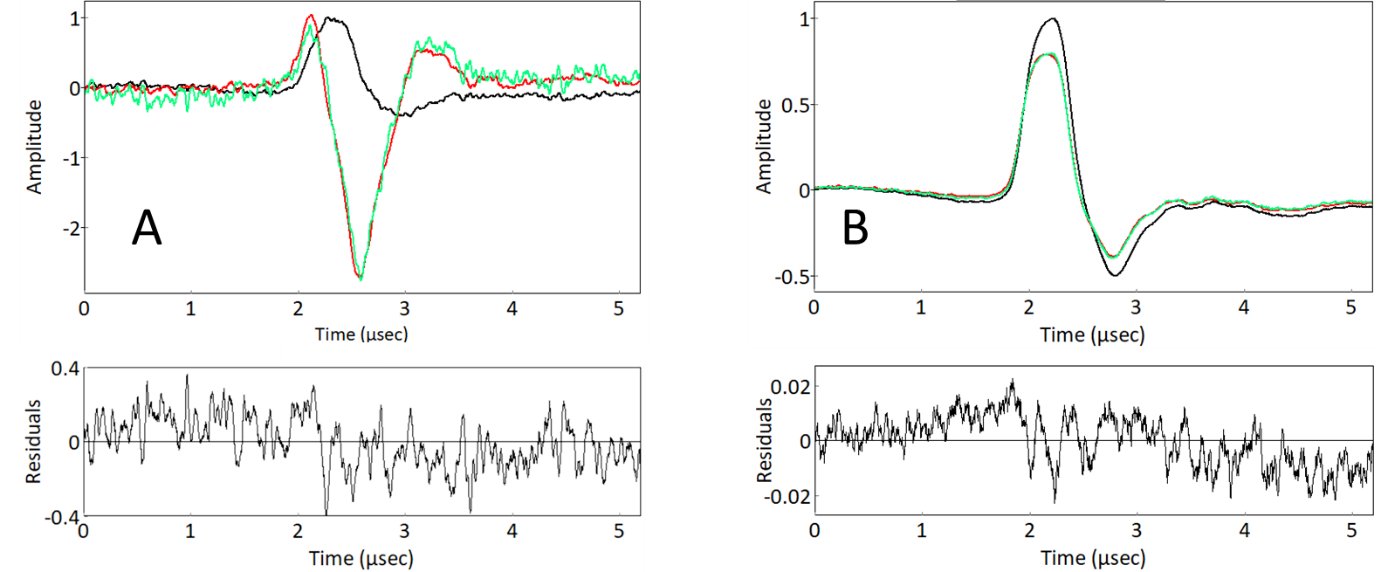
**

**Figure S1.** Representative deconvolution analysis of photoacoustic signals for GAF3 _G_ at T = 7°C (A) and T = 20 °C (B). Black solid curves are the IRF measured with a BBBN solution. Red solid curves are the signals from GAF3_G_. State GAF3_G_ was selected by cw illumination with the 514 nm output of an Ar ion laser. The solution was stirred to ensure homogeneity. The green solid lines are the reconvolution of the IR signals with a biexponential decay.


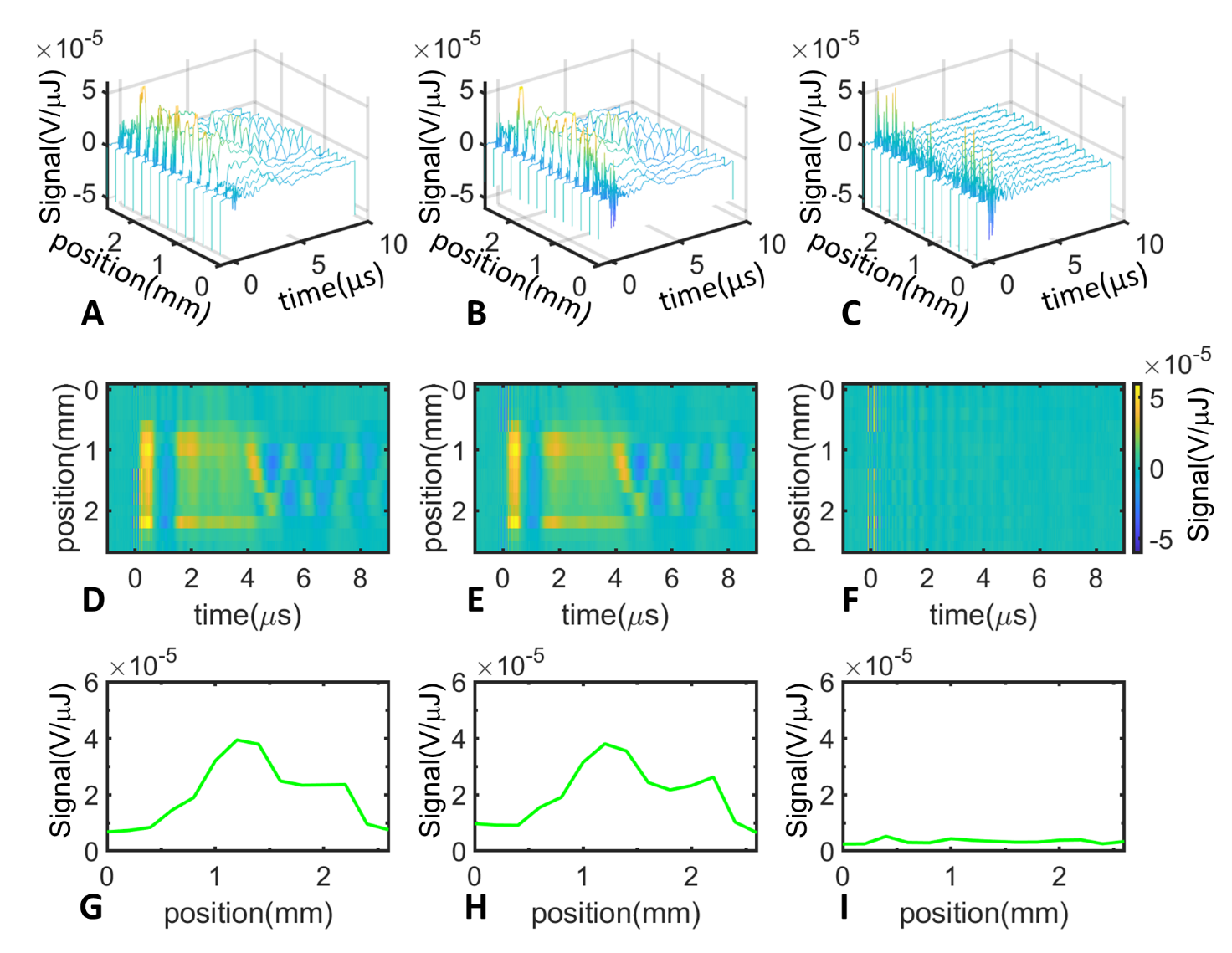


**Figure S2.** Photoacoustic signals collected for a capillary tube filled with a transformed *E. coli* (overexpressing GAF3) bacterial suspension immersed in a water filled cuvette. Pulsed excitation was at 540 nm, T = 20 °C. The top row (A-C) reports waterfall plots of the photoacoustic signals as a function of time and of the position of the excitation beam inside the cuvette for GAF3_R_ (A), GAF3_G_ (B), and the GAF3_G_ - GAF3_R_ difference (C). The center row (D-F) shows the contour plots for the signals in (A-C). The bottom row (G-I) shows the amplitude of the first positive oscillation of the PA signals in (A-C) as a function of the excitation beam position inside the cuvette.


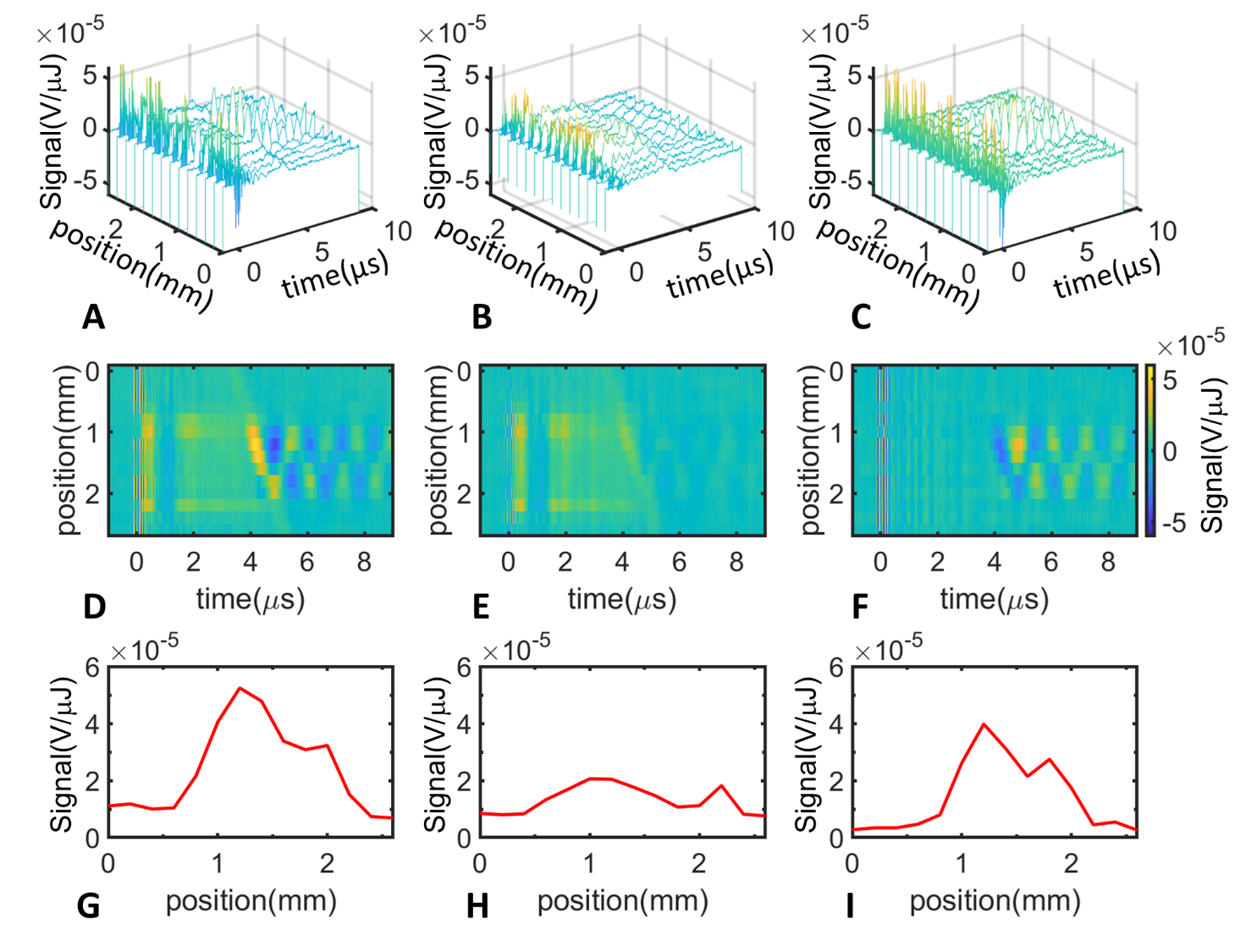


**Figure S3.** Photoacoustic signals collected for a capillary tube filled with a transformed *E. coli* (overexpressing GAF3) bacterial suspension immersed in a water filled cuvette. Pulsed excitation was at 650 nm, T = 20 °C. The top row (A-C) reports waterfall plots of the photoacoustic signals as a function of time and of the position of the excitation beam inside the cuvette for GAF3_R_ (A), GAF3_G_ (B), and the GAF3_R_ - GAF3_G_ difference (C). The center row (D-F) shows the contour plots for the signals in (A-C). The bottom row (G-I) shows the amplitude of the first positive oscillation of the PA signals in (A-C) as a function of the excitation beam position inside the cuvette.


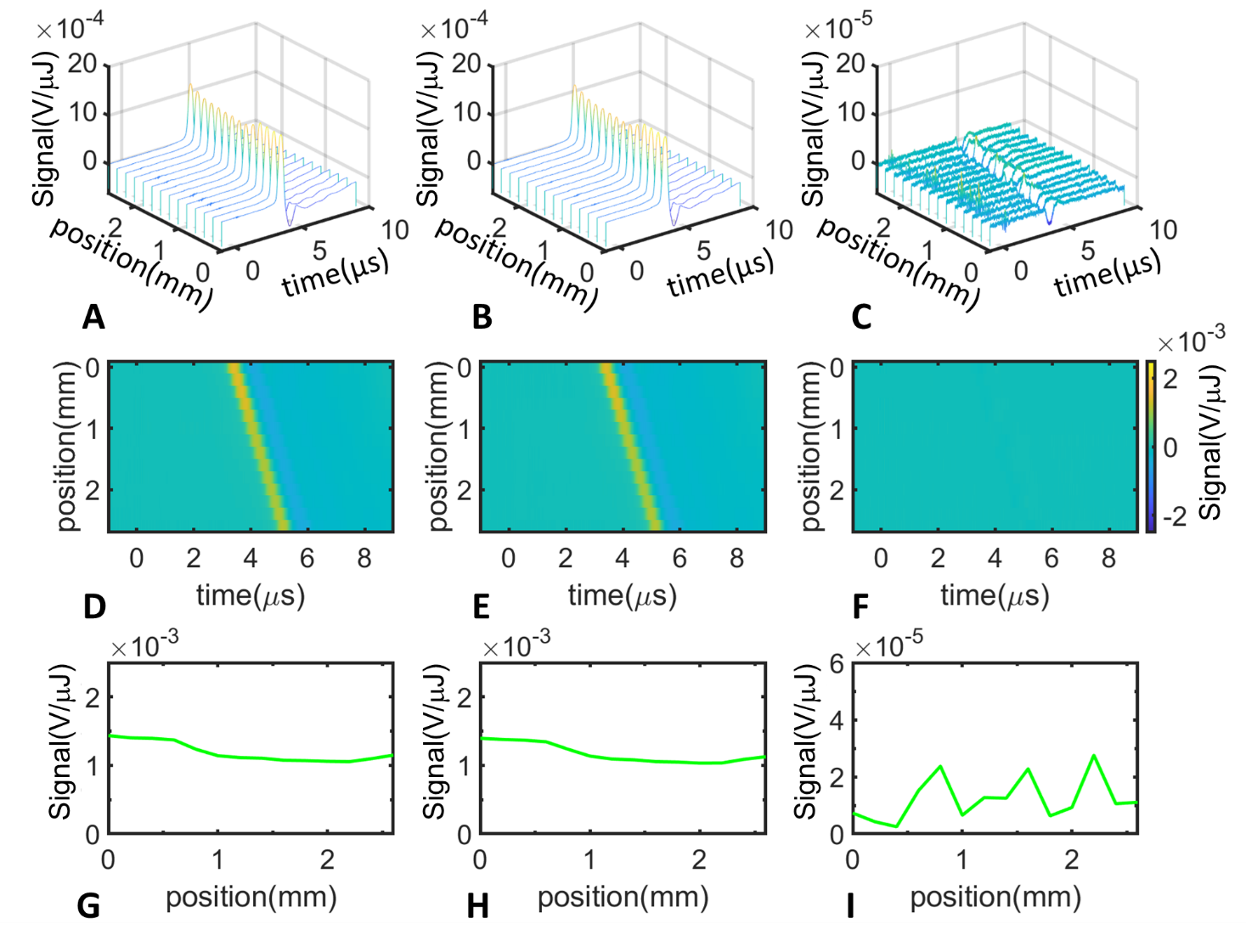


**Figure S4.** Photoacoustic signals collected for a capillary tube filled with a transformed *E. coli* (overexpressing GAF3) bacterial suspension immersed in a cuvette filled with a BBBN solution, A(540 nm) = 0.5 . Pulsed excitation was at 540 nm, T = 20 °C. The top row (A-C) reports waterfall plots of the photoacoustic signals as a function of time and of the position of the excitation beam inside the cuvette for GAF3_R_ (A), GAF3_G_ (B), and the GAF3_G_ - GAF3_R_ difference (C). The center row (D-F) shows the contour plots for the signals in (A-C). The bottom row (G-I) shows the amplitude of the first positive oscillation of the PA signals in (A-C) as a function of the excitation beam position inside the cuvette.


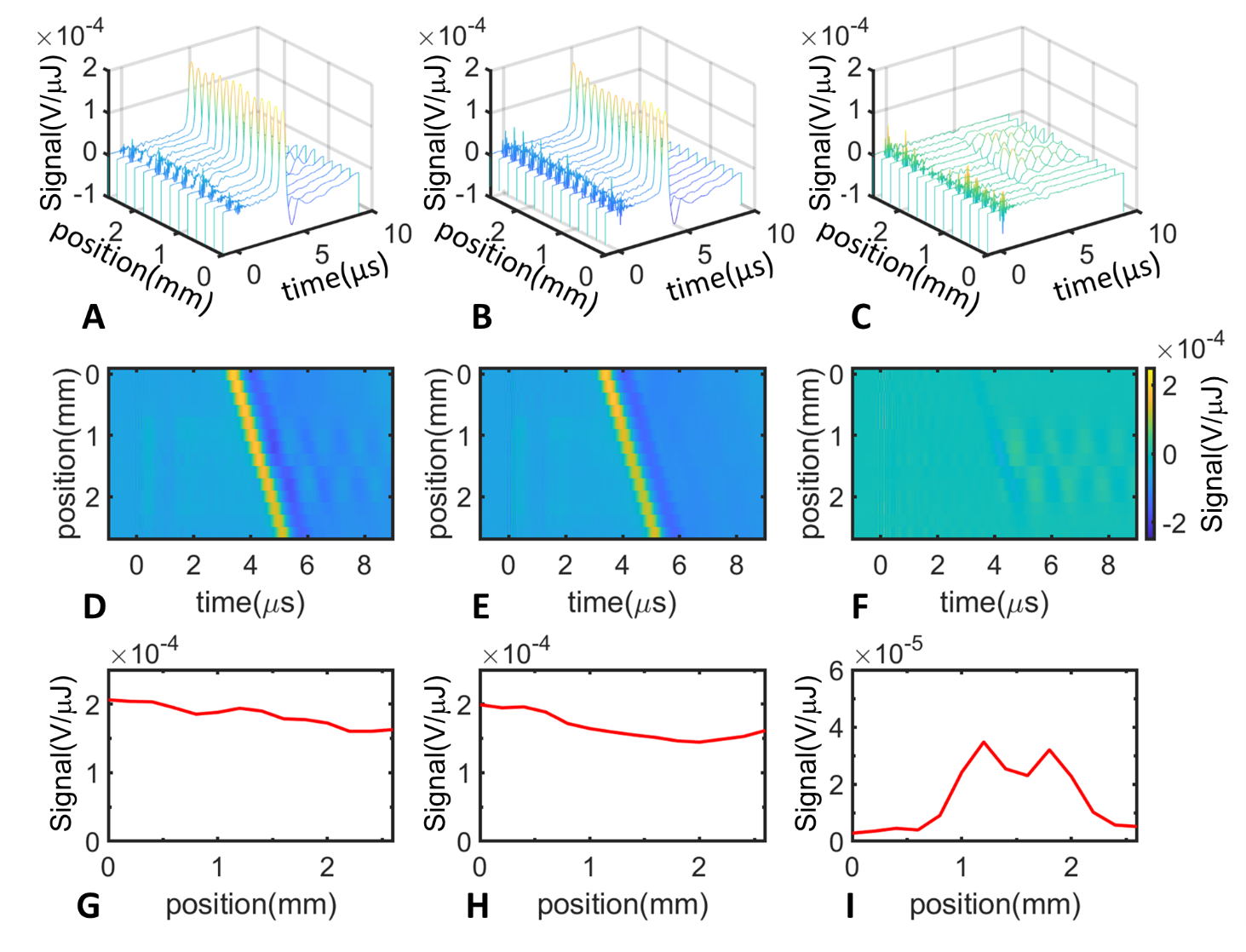


**Figure S5.** Photoacoustic signals collected for a capillary tube filled with a transformed *E. coli* (overexpressing GAF3) bacterial suspension immersed in a cuvette filled with a BBBN solution, A(650 nm) = 0.75. Pulsed excitation was at 650 nm, T = 20 °C. The top row (A-C) reports waterfall plots of the photoacoustic signals as a function of time and of the position of the excitation beam inside the cuvette for GAF3_R_ (A), GAF3_G_ (B), and the GAF3_R_ - GAF3_G_ difference (C). The center row (D-F) shows the contour plots for the signals in (A-C). The bottom row (G-I) shows the amplitude of the first positive oscillation of the PA signals in (A-C) as a function of the excitation beam position inside the cuvette.


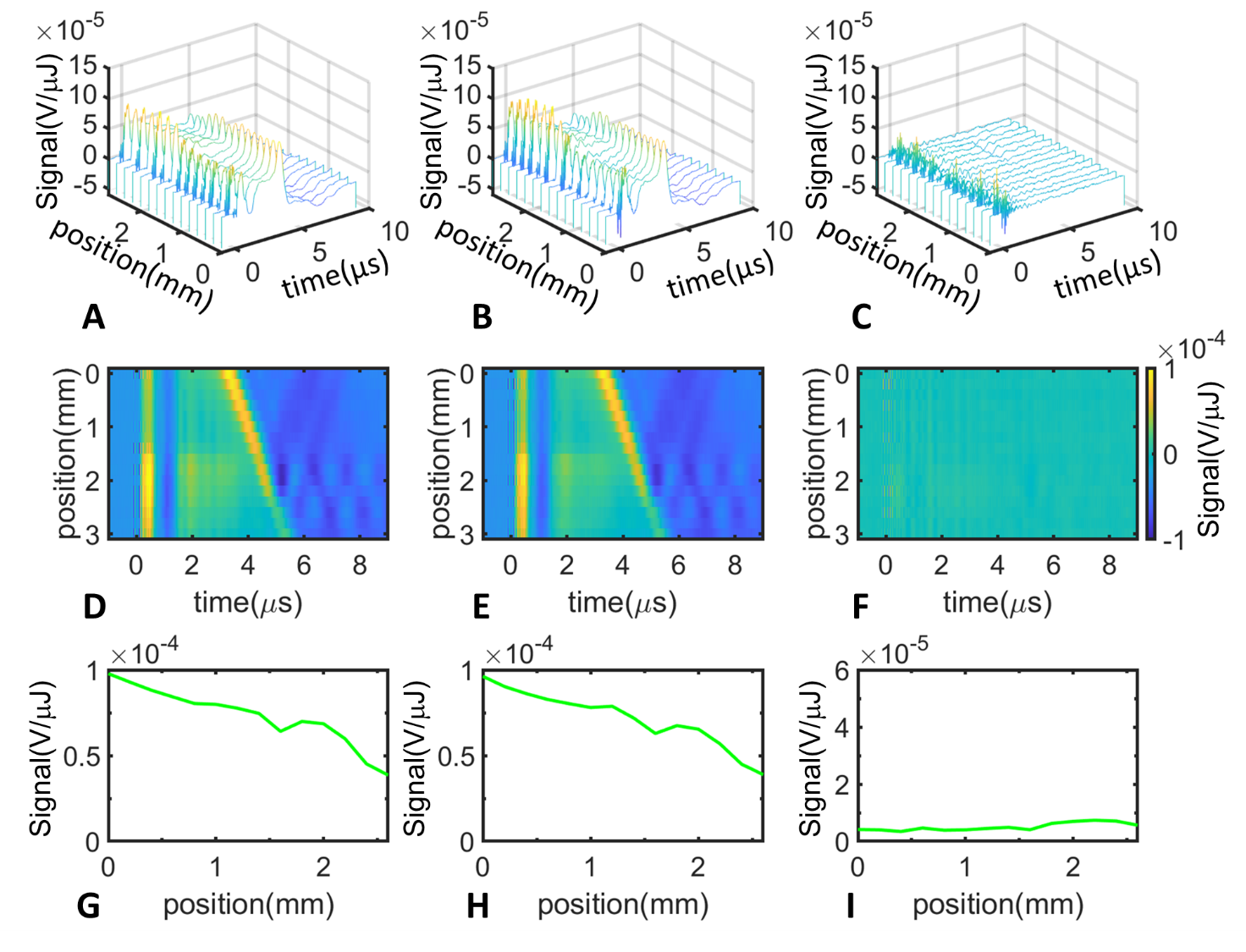


**Figure S6.** Photoacoustic signals collected for a capillary tube filled with a transformed *E. coli* (overexpressing GAF3) bacterial suspension immersed in a cuvette filled with an *E. coli* suspension, A(600 nm) = 0.5. Pulsed excitation was at 540 nm, T = 20 °C. The top row (A-C) reports waterfall plots of the photoacoustic signals as a function of time and of the position of the excitation beam inside the cuvette for GAF3_R_ (A), GAF3_G_ (B), and the GAF3_R_ - GAF3_G_ difference (C). The center row (D-F) shows the contour plots for the signals in (A-C). The bottom row (G-I) shows the amplitude of the first positive oscillation of the PA signals in (A-C) as a function of the excitation beam position inside the cuvette.


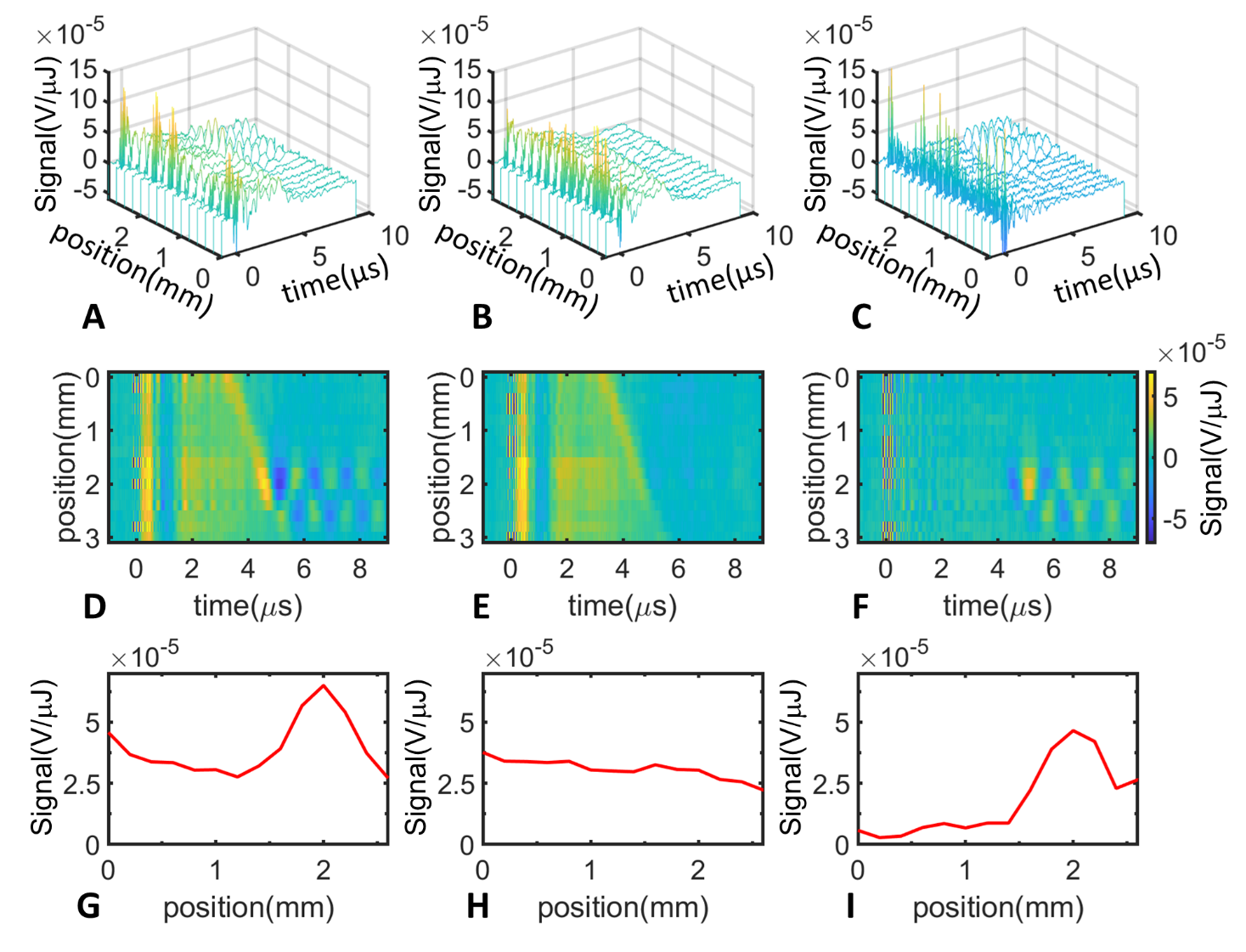


**Figure S7.** Photoacoustic signals collected for a capillary tube filled with a transformed *E. coli* (overexpressing GAF3) bacterial suspension immersed in a cuvette filled with an *E. coli* suspension, A(600 nm) = 0.5 . Pulsed excitation was at 650 nm, T = 20 °C. The top row (A-C) reports waterfall plots of the photoacoustic signals as a function of time and of the position of the excitation beam inside the cuvette for GAF3_R_ (A), GAF3_G_ (B), and the GAF3_R_ - GAF3_G_ difference (C). The center row (D-F) shows the contour plots for the signals in (A-C). The bottom row (G-I) shows the amplitude of the first positive oscillation of the PA signals in (A-C) as a function of the excitation beam position inside the cuvette.
